# New Mutation of Pelizaeus­-Merzbacher-Like Disease; A Report from Iran

**DOI:** 10.5812/iranjradiol.6913

**Published:** 2014-05-15

**Authors:** Parvaneh Karimzadeh, Farzad Ahmadabadi, Omid Aryani, Massoud Houshmand, Alireza Khatami

**Affiliations:** 1 Pediatric Neurology Research Center, Shahid Beheshti University of Medical Sciences, Tehran, Iran; 2 Ardabil University of Medical Sciences, Ardabil, Iran; 3 Special Medical Center, Tehran, Iran; 4 Department of Human Genetics, National Institute for Genetic Engineering and Biotechnology, Tehran, Iran

**Keywords:** Pelizaeus­Merzbacher-Like Disease, Neurodegenerative Disease Leukodencephalopathy, Children

## Abstract

Pelizaeus­-Merzbacher-like disease (PMLD) is a hypomyelinating leukoencephalopathy disorder with a genetically heterogeneous pattern. Mutations in the GJA12/GJC2 gene cause one form of autosomal recessive Pelizaeus­-Merzbacher-like disease. Here, we report a new mutation in a ­10-month-old girl with nystagmus, psychomotor delay, hypotonicity, head nodding and dysmyelination from healthy second cousin parents. The genetic study showed a homozygote deletion as c902-918del in the exone 2. According to our study and recent reports from other Middle East countries, we suggest GJA12 gene mutations are common in this area, but we didnot find any previous report about this new mutation (c902-918Del).

## 1. Introduction

Pelizaeus Merzbacher disease (PMD) is a progressive disorder in myelin formation that is transmitted with an X­-linked pattern of inheritance. This disease was initially described in 1885 by Pelizaeus and in 1910 by Merzbacher. In 1964, Zeman and colleagues explained the role of proteolipid protein (PLP1) in this disease. The gene of this disease is located on the long arm of the X chromosome (Xq21.2­q-22) (1). Dysmyelination is the major pathologic defect in PMD. The MRI pattern is highly suggestive of the disease. It shows arrest of myelination in the stage that the brain should be myelinated. There is a correlation between the pattern of myelinated white matter and the clinical severity of the disease. The T1 weighted images show low intensity of all unmyelinated white matter structures; whereas, these structures have high signal intensity in T2 (2). Mutation in PLP1 that encodes the essential intrinsic membrane protein of CNS myelin is the main cause of PMD (1).

PMD is an X-linked myelin synthesis disorder that causes neurodevelopmental delay and hypotonicity in infancy (3). Symptoms initially begin before 3 months of age and gradually seizure and optic atrophy appear. There is diffuse white matter involvement in the brain MRI (1). The aggressive form of the disease is presented with severe hypotonia at birth. These patients have stridor and feeding difficulties and are very similar to spinal muscular atrophy (SMA) without any involvement in anterior horn cells. Spastic paraplegia type 2 (X-linked type of spastic paraplegia) is an allelic variant of PMD with PLP gene defect that presents with spasticity in the lower extremities and slow progression (4). Pelizaeus­Merzbacher­like disease (PMLD) is clinically similar to PMD and include nystagmus, ataxia and hypotonicity followed by spasticity. This is a disease that is identical to PMD, but mutation of the PLP1 gene is not detected. In most cases, no gene has been identified, but in a small subset of them (less than 10%) mutation in GJC2 (so called GJA12) codon for Connexin 46.6 (Cx47) has been found (3).

This gene is called GJA12 and encodes the gap junction protein 12. Mutation of the gap junction of protein alpha 12 can cause one of the autosomal recessive types of PMLD (5). Diagnosis is performed by sequencing the entire coding region of GJC2. This assay will detect point mutation, small deletion and small insertion. MRI shows diffuse white matter involvement and basal ganglia calcification.

## 2. Case Presentation

A 10-month-old girl was referred for evaluation of neurodevelopmental delay. She was the product of the first pregnancy of consanguineous parents (second cousins), born full term by cesarean section without any history of birth problems (such as asphyxia, hypoxia, icter and hypoglycemia). Her birth weight was 2700 gr and head circumference was 35 cm.

In physical and neurological examination, motor development evaluation showed the ability of rolling, but head control was incomplete. Head nodding was seen. She had hypotonicity and in vertical suspension, she did not have weight bearing. Pendular bilateral nystagmus was significant since neonatal period. There was no history of seizure.

**Figure 1.**
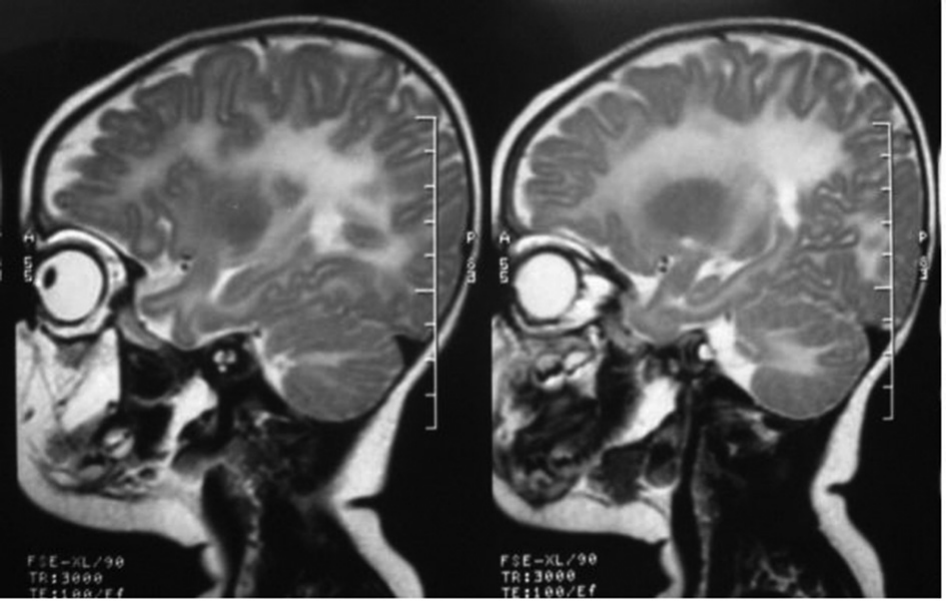
A ten-month-old girl with developmental delay. Sagittal T2 weighted MRI reveals diffuse increased signal intensity in the cerebral and cerebellar white matter. U fibers are also involved.

**Figure 2.**
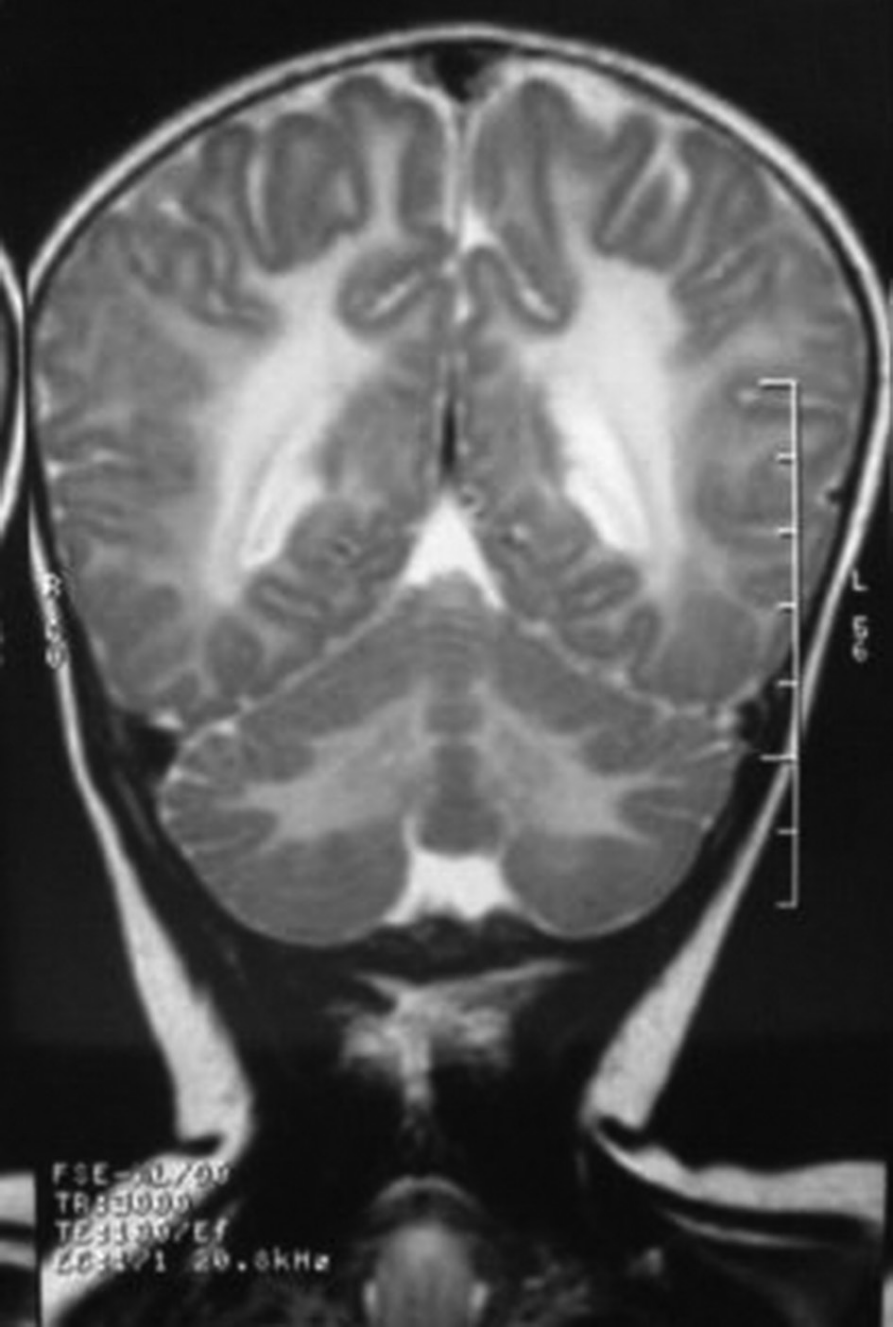
Coronal T2 weighted MRI shows diffuse increased signal intensity in the white matter, in the supra- and infratentorial regions. Symmetric increased signal intensity is seen in both hemispheres.

**Figure 3.**
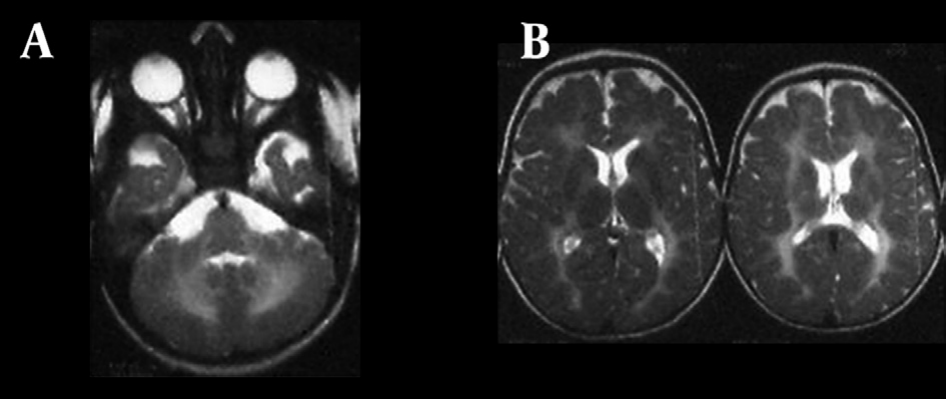
A) Axial T2 weighted image reveals involvement of the cerebellar white matter symmetrically; B) Axial section at upper levels reveals symmetrical increased signal intensity in the cerebral white matter and bilateral globus pallidus.

In paraclinic evaluation, MRI showed abnormal signal intensity in the white matter (hyperintensity in T2, FLAIR and low signal intensity in T1 weighted images). It showed an arrest of myelination and this image is compatible with a younger chronological age. Abnormal findings included diffuse hypersignal white matter in the cerebral and cerebellar white matter, genu of the corpus callosum, basal ganglia especially globus pallidus and ventrolateral thalamic nuclei (Figures 1, 2 and 3). Neurometabolic study was in normal limits and EEG showed no significant changes. VEP showed increased latency (P100=138).

In genetic study, genomic DNA was isolated from blood cells, using the QIA GEN extraction kit (Zist Baran) (5). Oligonucleotide primers were used to PCR-amplify the single coding exon of GJA12/GJC2 from genomic DNA in three overlapping fragments. This sequencing was previously described by Bugiani (6). PCR conditions were as follows: 100 ng of genomic DNA was amplified in a total volume of 10 μL, containing 0.5 μL 5 mM dNTP, 50 ng of each primer, 5 μL 2×GC-I buffer (Sinagene, Iran), and 1 μL of TaqDNA polymerase (Sinagene, Iran).

The reaction program was 94˚C for 5 minutes, then 34 cycles at 94˚C for 1 minute, 58˚C for 15 seconds, and 72˚C for 1 minute, followed by a final extension step of 5 minutes at 72˚C. The PCR products were purified and sequenced using either sense or antisense primer by a BigDye terminator cycle sequencing kit in the ABI PRISM 3730 genetic analyzer (PE Applied Biosystems, Foster City, CA). PCR products were directly sequenced using an ABI PRISM, 3730 DNA Analyzer and BigDye Terminator Cycle Sequencing Kit version 1.1, according to the protocol of the manufacturer (Applied Biosystems). FinchTV and NCBI programs were used for analyzing the DNA sequences.

## 3. Discussion

Whole exon sequencing of PLP1 gene showed normal sequences in the patient’s blood sample. A homozygote deletion c902­-918 del in exon2 of GJA12 was found by the sequencing method. This frame shift deletion changed the amino acid frame in GJA12 gene.

PMLD is described clinically as PMD, but there is no mutation in the PLP1 gene that causes PMD. Both PMD and PMLD are characterized by rapid nystagmus, psychomotor delay, ataxia, and difficult articulation (3). Nystagmus is detected by 7 weeks and motor delay is noted by 15 months of age (6). Mutation of the gap junction protein alpha12 (GJA12) gene is known as one of the autosomal recessive PMLD forms. Few patients with the mutation of GJA12 have been reported. Therefore, GJA12 mutations seem rare causes of PMLD. Their clinical phenotypes are overall compatible with the clinical manifestations of the mild forms of PLP1-related disorders, but better cognition and earlier signs of axonal degeneration is prominent (5). On the other hand, some of PMLD cases are transmitted by an X­-linked pattern. Gene locus is located on the outer part of the PLP gene location on the X chromosome. Another form of PMLD is expressed as autosomal recessive mutations in GJA12 that encodes gap junction protein (Connexin 46.6). This gene encodes Connexin 46.6 that has a cardinal role in central and peripheral myelination. Their phenotypes include nystagmus, developmental delay and progressive spasticity.

In 2004, Orthmann and colleagues mentioned a form of PMLD in a Turkish family that was transmitted by an autosomal recessive pattern (7). These patients had nystagmus, developmental delay, ataxia, dystonia, dysarthria and progressive spasticity. These clinical manifestations were presented in early infancy. They had also mild peripheral sensory and motor neuropathy. Five different mutations in GJA12 gene were detected in these patients (7). Bugiani reported eight members of a Saudi Arabia family with PMLD. MRI of the patients showed diffuse white matter involvement (6). Henneke and colleagues in 2008 reported 182 families with the diagnosis of PMLD. In 16 cases (of 14 families), 11 mutations in the GJA12 gene were detected. They studied the mutations of GJA12 and reported that this mutation is the rare cause of PMLD (5).

Based on prior studies on PMLD (2004), mutation in the GJA12 gene was the rare cause of this disease (9). But since 2006, when many cases were reported from the Middle East, it seems that this proportion has changed (5, 8). One of the other forms of PMLD is due to mutation in the thyroid hormone transporter gene MCT. These patients have PMD clinical manifestations without progressive dysmyelination patterns. The importance of assessment of free T3 and T4 levels in PMLD patients younger than 3 years of age has been explained by these results (9). 

Our patient had a mutation in GJA12 gene and her clinical findings were very similar to other PMLD cases. She had a new mutation (c902-918Del) that had not been reported in previous studies. With respect to our case and recent reports from other Middle East countries, we think GJA12 gene mutations are common in this area, but we found a new mutation (c902­-918 Del); our study has evaluated and reported this mutation.
